# Teaching Microsurgical Breast Reconstruction—A Retrospective Cohort Study

**DOI:** 10.3390/jcm10245875

**Published:** 2021-12-14

**Authors:** Sebastian Fischer, Yannick F. Diehm, Dimitra Kotsougiani-Fischer, Emre Gazyakan, Christian A. Radu, Thomas Kremer, Christoph Hirche, Ulrich Kneser

**Affiliations:** Department of Hand, Plastic and Reconstructive Surgery, Burn Center, Hand and Plastic Surgery of Heidelberg University, BG Clinic Ludwigshafen, Ludwig-Guttmann-Str. 13, 67071 Ludwigshafen, Germany; sebastian.fischer@bgu-ludwigshafen.de (S.F.); yannick.diehm@bgu-ludwigshafen.de (Y.F.D.); dimitra.kotsougiani@bgu-ludwigshafen.de (D.K.-F.); emre.gazyakan@bgu-ludwigshafen.de (E.G.); christian.radu@bgu-ludwigshafen.de (C.A.R.); thomas.kremer@sanktgeorg.de (T.K.); christoph.hirche@bgu-frankfurt.de (C.H.)

**Keywords:** breast reconstruction, deep inferior epigastric perforator flap, transverse myocutaneous gracilis flap, learning curve, training

## Abstract

Microsurgical breast reconstruction demands the highest level of expertise in both reconstructive and aesthetic plastic surgery. Implementation of such a complex surgical procedure is generally associated with a learning curve defined by higher complication rates at the beginning. The aim of this study was to present an approach for teaching deep inferior epigastric artery perforator (DIEP) and transverse upper gracilis (TUG) flap breast reconstruction, which can diminish complications and provide satisfying outcomes from the beginning. DIEP and TUG flap procedures for breast reconstruction were either performed by a senior surgeon (>200 DIEP/TUG, ”*no-training* *group*”), or taught to one of five trainees (>80 breast surgeries; >50 free flaps) in a step-wise approach. The latter were either performed by the senior surgeon, and a trainee was assisting the surgery (“*passive training*”); by the trainee, and a senior surgeon was supervising (“*active training*”); or by the trainee without a senior surgeon (“*after training*”). Surgeries of each group were analyzed regarding OR-time, complications, and refinement procedures. A total of 95 DIEP and 93 TUG flaps were included into this study. Before the first DIEP/TUG flap without supervision, each trainee underwent a mean of 6.8 DIEP and 7.3 TUG training surgeries (*p* > 0.05). Outcome measures did not reveal any statistically significant differences (passive training/active training/after training/no-training: OR-time (min): DIEP: 331/351/338/304 (*p* > 0.05); TUG: 229/214/239/217 (*p* > 0.05); complications (n): DIEP: 6/13/16/11 (*p* > 0.05); TUG: 6/19/23/11 (*p* > 0.05); refinement procedures (n): DIEP:71/63/49/44 (*p* > 0.05); TUG: 65/41/36/56 (*p* > 0.05)), indicating safe and secure implementation of this step-wise training approach for microsurgical breast reconstruction in both aesthetic and reconstructive measures. Of note, despite being a perforator flap, DIEP flap required no more training than TUG flap, highlighting the importance of flap inset at the recipient site.

## 1. Introduction

Improved awareness, early detection through screening, and treatment advances have significantly increased survival rates of breast cancer and concomitant number of patients seeking for breast reconstruction [1,2]. Nowadays, autologous techniques for breast reconstruction can provide convincing outcomes and high patient satisfaction and thus a real alternative to traditional implant-based approaches [3,4,5,6]. In particular, results after free deep inferior epigastric artery perforator (DIEP) flap or, in patients with less abdominal tissue, after transverse upper gracilis (TUG) flap, are favorable regarding donor site morbidity, reliability and aesthetics, making both flaps workhorses in microsurgical breast reconstruction [7,8,9,10]. Compared to silicone implant-based reconstructions, autologous tissue obtains several advantages, of which the most important for oncologic patients is the significantly lower complication rate after irradiation therapy [11]. In this case, some authors even suggest autologous tissue transfer as reconstruction of choice, if donor sites are available [12,13]. Nevertheless, in current clinical practice, an imbalance in favor of implant-based breast reconstruction is still a reality, being applied in more than 80% of reconstructive cases [2]. One problem is that clinical implementation of such a complex surgical procedure can be very difficult and risky, both for the patient and economically [14,15,16,17,18]. Specific skills and experience are required for solid and satisfying outcomes. Several studies already investigated the implementation process of microsurgical breast reconstruction and described a so-called learning curve [19,20,21]. This learning curve consists of three distinct phases, namely, the beginning point; the segment reflecting the rate of learning; and the expert level, where performance plateaus and stable outcomes are reached. Ideally, the second phase, which usually involves the highest rate of complications, is shortened or even omitted completely. Recent studies demonstrated that a learning curve in microsurgical breast reconstruction could be bypassed if the surgeon underwent an adequate training [22]. Indeed, learning required skills and gathering experience under supervision could avoid complications and thus diminish the learning curve [22]. However, requirements for and the process of such training remain unclear. Details about teaching microsurgical breast reconstruction could ease and improve the implementation process and thus broaden its application. Furthermore, an appropriate training concept would improve patient safety and outcomes.

The aim of this study was to present an approach for teaching deep inferior epigastric artery perforator (DIEP) and transverse upper gracilis (TUG) flap breast reconstruction, which can diminish early complications and provide secure and satisfying outcomes from the first breast reconstruction with DIEP or TMG flap, respectively.

## 2. Materials and Methods

After institutional review board approval (protocol no. 837.516.16(10834)), medical records of all patients, who underwent microsurgical breast reconstruction within a three-year-period (October 2015–September 2018) at our institution, were reviewed for this study. Unilateral autologous tissue transfers by means of free DIEP or TUG flaps were included. Patients with prior flap loss were excluded from the study. Besides the type of breast reconstruction, former irradiation or chemotherapy, OR time, revision surgeries due to vascular complications, hematoma, and other complications as well as secondary procedures for aesthetic purposes (refinement procedures, such as nipple areolar reconstruction, contralateral mastopexy/reduction, lipofilling, scar correction, resection of fat necrosis and oil cysts) were documented. Furthermore, microsurgical experience of each surgeon gathered before the beginning of breast reconstruction training was analyzed. The name “trainee” was chosen to indicate the participation in this microsurgical breast reconstruction training program and should not be confused with plastic surgery training during residency. All participants of the introduced training program have completed their residency and were board-certified plastic surgeons. 

### 2.1. Training Concept

Each of the five trainees underwent an individual number of training surgeries, in which the trainee was taught by one of two established senior reconstructive breast surgeons. Besides board certification, requirement to take part in this microsurgical breast reconstruction training program as a trainee was sufficient experience in general breast surgery (>80 breast surgeries as active surgeon; procedures such as breast lifts, breast reductions, breast augmentations, or corrections of tubular breasts), as well as in microsurgery (>50 free flaps as active surgeon; free flaps such as such as anterior lateral thigh, latissimus dorsi, parascapular, rectus abdominis, and gracilis). In the selected cohort of trainees in this study, each trainee performed 115.4 (range 81 to 186) general breast surgeries, and 105.4 free flaps on average (range 52 to 198) before starting the training program. Furthermore, each trainee was participating in a vast number of microsurgical breast reconstruction cases during their residency and was familiar with the current literature of microsurgical breast reconstruction. All trainees completed the majority of their residency in our clinic. 

Training surgeries were further divided into passive and active training surgeries. While during passive training, the trainee watched the senior surgeon performing the surgery, active training implied operating by the trainee under supervision of the senior surgeon. In other words, passive training was assisting the senior surgeon while he was operating the flap, and active training was operating the flap while the senior surgeon was assisting. As soon as the senior surgeon decided that the trainee was sufficiently skilled and experienced, this fact was discussed in the senior surgeon team, and a formal decision was made that training was completed and subsequent surgeries should be performed without supervision. However, each procedure was monitored by a senior surgeon with regard to complications and reconstructive results. Moreover, a senior surgeon was on standby and able to participate in the procedure, if needed. Surgeries for active and passive training as well as surgeries performed by the trainee after completed training were compared to each other and to a “no-training” group, in which both senior surgeons operated without teaching any of the trainees. 

### 2.2. Statistical Analysis

Differences in operation time between groups were analyzed with one-way analysis of variance (ANOVA) followed by Bonferroni’s correction for posttest corrections. The chi squared test was used to compare complications rates between groups. Data are expressed as percentages and means with their standard deviation (SD). A *p*-value ≤ 0.05 was considered significant. All analyses were performed using GraphPad Prism Version 7.0c (GraphPad Software Inc., La Jolla, CA, USA).

### 2.3. Standard Surgical Technique

Preoperatively, all patients for DIEP flap breast reconstruction underwent computed tomography angiography (CT-A) for inferior epigastric perforating vessels. The flap design based on the intraoperative flap viability and perfusion were depicted by indocyanine green fluorescence angiography. Recipient vessels were internal mammary artery and vein in all cases. No internal mammary vessel perforators were used. Perforator diameter was about 1.4 to 2.2 mm in all DIEP cases. In most of the cases, medial row perforators were chosen. Approximately one-fifth of perforators were lateral perforators with long course. No extramuscular perforators were found in this study. 3.5× magnification was used for perforator dissection. During surgery, mono- and bipolar cautery were used, and for perforator dissection and branch ligature, a Ligaclip clip applier was utilized. The intraoperative decision regarding perforator selection was based on the size of the perforator, comtantes veins, and preoperative CT-A. In addition, selective clamping during ICG angiography was performed intraoperatively, and the perfusion pattern was analyzed for both perforator selection and selection of the flap design. All procedures were performed in a two-team approach. One team consisting of a senior resident performed the dissection of the internal mammary vessels, as well as, if necessary, implant removal and pocket preparation. In case of direct reconstruction (9/96 DIEPs, 42/93 TUGs), a gynecologist undertook the mastectomy. The other team (trainer plus trainee) harvested the flap according to established standard techniques. Once the flap was elevated, the same team inset the flap and performed the arterial anastomosis with interrupted sutures (8-0 Ethilon, Ethicon Inc., Sommerville, NJ, USA). A coupler device (Synovis Micro Companies Alliance Inc., Birmingham, AL, USA) was used for venous anastomosis. Two venous anastomoses were performed whenever possible in DIEP flaps. Flap inset at the breast was based on mastectomy pattern, flap size, size and shape of the contralateral breast, and individual preferences of the surgeon. Postoperative flap monitoring consisted of capillary refill assessment every hour for 5 days. Importantly, the same operative technique and postoperative protocol for flap monitoring and anticoagulation was utilized in all cases.

## 3. Results

Within the study period, we performed 96 DIEP and 93 TUG flaps for unilateral microsurgical breast reconstruction. Subgroup separation into “no training”, “passive training”, “active training”, and “after training” revealed 18, 17, 24, and 37 DIEP and 27, 17, 27, and 22 TUG flaps, respectively. Before the first DIEP flap without supervision, each trainee underwent a mean of 2.8 passive (range 2 to 4) and 4.0 (range 2 to 7) active DIEP training surgeries. Regarding TUG flaps, a mean of 2.8 passive (range 2 to 7) and 4.5 active (range 2 to 9) training surgeries were necessary.

Epidemiologic data, previous oncologic therapy of each patient, and number of training procedures are given in Table 1.

**Figure 1:**
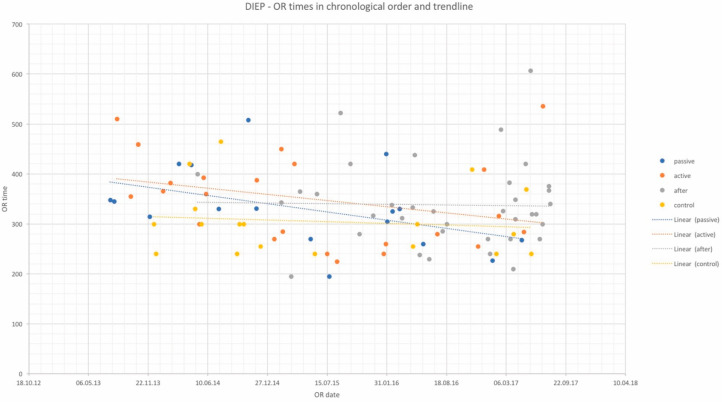
OR times of DIEP flap procedures in chronological order and trendlines of each study group. *x*-axis: date of surgery; *y*-axis: OR time in minutes.

**Figure 2:**
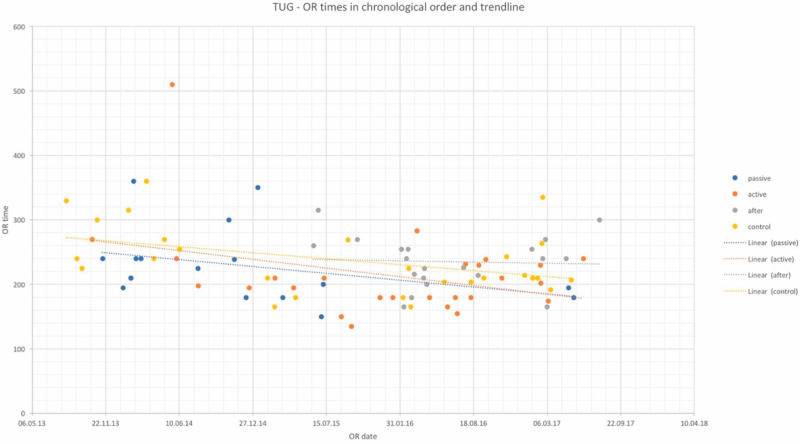
OR times of TUG flap procedures in chronological order and trendlines of each study group. *x*-axis: date of surgery; *y*-axis: OR time in minutes.

**Figure 3:**
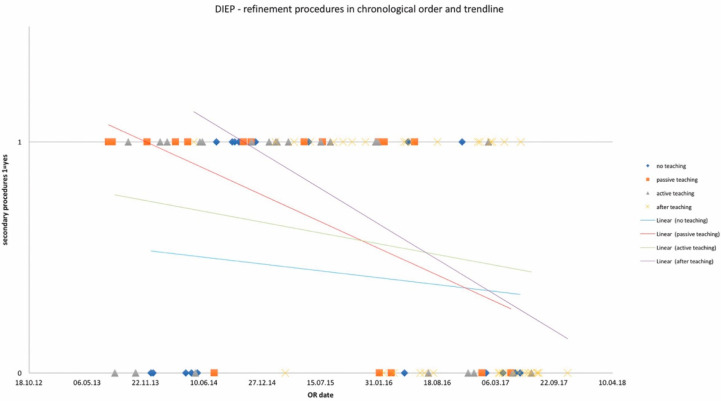
Number of refinement procedures in chronological order and trendlines of each study group. *x*-axis: date of surgery; *y*-axis: incidence of refinement procedure.

**Figure 4:**
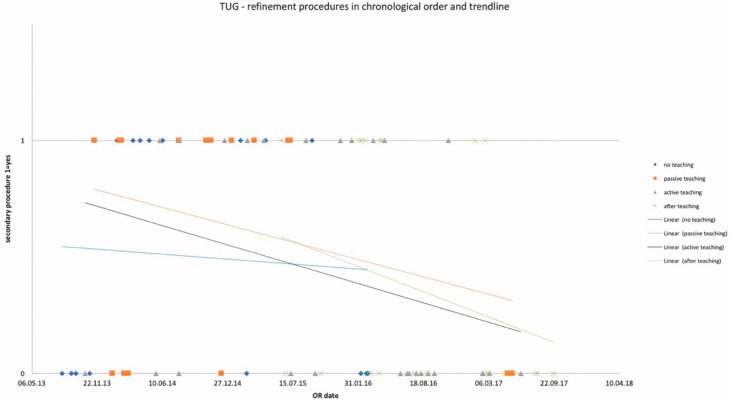
Number of refinement procedures in chronological order and trendlines of each study group. *x*-axis: date of surgery; *y*-axis: incidence of refinement procedure.

OR times did not differ significantly among study groups and are depicted in Table 2 for DIEP and Table 3 for TUG flaps, as well as in chronological order with trend in Figure 1 and Figure 2. Complication rates for DIEP and TUG flap procedures are shown in Table 2 and Table 3, respectively. Half of the OR time was spent with the dissection of perforators, one-quarter for anastomosis, and the remaining quarter for insetting of the flap. Complete flap loss was seen in two cases—one TUG of the “*active training*” group and one DIEP of the “*after training*” group. Partial flap loss was seen in four cases—one DIEP and one TUG flap in each “*passive training*” group, one DIEP flap in the “*after training*” group, and one TUG in the “*no training*” group. Among study groups, vascular revisions, other complications, and secondary procedures did not reveal any statistically significant differences. For TUG, refinement procedures were necessary in 65%, 41%, 36%, and 56% of cases in the passive, active, after-training, and no-training groups, respectively. In the DIEP groups, patients underwent refinement procedures in 71%, 63%, 49%, and 44% of cases in the passive, active, after-training, and no-training groups, respectively. Number of refinement procedures are listed in Table 2 and Table 3. Refinement procedures in chronological order with trend line are shown in Figure 3 and Figure 4. Of note, breast reduction/mastopexy of the contralateral side and reconstruction of the nipple areola complex were always done in a second procedure and counted as a refinement procedure.

There was no correlation between the prior microsurgical experience, i.e., number of free flap procedures, and the achieved outcomes of each trainee.

## 4. Discussion

This study demonstrates that microsurgical breast reconstruction can be taught safely and securely by means of a stepwise training approach. After training a mean of 6.8 and 7.3 DIEP and TUG flaps, respectively, surgeons with significant microsurgical experience but not familiar with microsurgical breast reconstruction were able to perform both free flaps autonomously and with reliable outcomes. Complication rates, number of revisions, and number of secondary procedures did not vary from no-training group, in which surgeries were performed by experienced breast surgeons. Of note, DIEP flap breast reconstruction did not require significantly more teaching cases compared to reconstruction with non-perforator TUG flap, highlighting the importance of the recipient site in breast reconstruction. 

In 2008, Hallock published outcomes of his first 30 muscle perforator flaps and described a learning curve with significantly higher incidence of complications at the beginning [20]. The author stated that no surgeon will implement a new technique without passing a learning curve, but steepness of this learning curve will depend on the individual as well as preparations such as cadavesr dissections and formal training courses. In contrast, Grinsell et al. could not find outcomes that support existence of a learning curve after analysis of 214 DIEP flaps for breast reconstruction [22]. However, concomitant to Hallock, the authors highlighted the importance of an adequate training beforehand since this is crucial to avoid early complications. The results of our study are well in accordance with these findings. With means of a stepwise teaching approach, surgeons with only limited experience in autologous breast reconstruction were able to avoid early complications and thus to omit the learning curve. Of note, during training, complication rates did not increase, showing not only an effective but foremost a safe and secure way of teaching. 

With respect to perforator flap breast reconstruction, several studies claimed that the major challenge is meticulous dissection and handling of the perforator [20,22]. Indeed, in our study, DIEP flaps required more OR time compared to TUG flaps in general, and teaching of DIEP flaps took longer compared to no-training DIEP cases, a trend that was not seen in TUG flaps. Furthermore, MS-TRAM flap was more often utilized in the active training group, while all other groups revealed comparable results. Although this indicates that teaching a perforator flap is more complex compared to non-perforator flaps, differences were not statistically significant, and number of required training cases did not vary between both flap types. Importantly, in addition to board certification and a minimum of 80 breast surgeries, trainees in this study were well experienced in microsurgery (105.4 free flaps on average), and feeling comfortable with perforator flaps from other donor sites was a requirement to start the training program. This diminishes most of the technical challenges related to perforator flap harvest for breast reconstruction and should be well considered before including trainees into a microsurgical breast reconstruction program. 

An important aspect of teaching surgical skills is to define the point where the trainee can proceed to the next level of training or successfully accomplish the training. In this study, assessment criteria involved microsurgical technique, intraoperative decision-making regarding perforator selection, and flap viability as well as flap inset at the breast site. While these skills are certainly mandatory to become confident with the procedure, defining the point of someone being confident is very subjective and relies on teaching experience. However, since revision or complication rates as well as refinement procedures did not differ significantly among study groups, subjective assessment was retrospectively proven right by objective data. 

Another important aspect that needs to be addressed is that our department is well-experienced in teaching microsurgical procedures and has a significant case load for microsurgical free flaps with more than 350 procedures per year. Hirche et al. reported about our experience in teaching free flaps to fifth-year residents and revealed comparable outcomes regarding complications and OR times [23]. Kotsougiani et al. showed reliable results after finger replantation performed by senior residents of our institution, indicating safety and suitability of this teaching approach if experienced microsurgeons are supervising [24]. In both studies, we highlighted the importance of case selection suitable for teaching. 

In this study, we again selected the cases thoroughly before trainees were taught by the experienced breast microsurgeon. Patients with high risk for complications, such as coagulopathy, severe co-morbidity, or history of free flap failure, underwent either reconstruction with silicone implants, or the procedure was performed by one of the senior surgeons. The latter occurred in three patients of this study, who were not operated as teaching cases but by the senior surgeons and were thus excluded from the study.

Complications after microsurgical breast reconstruction are well described in the current literature and were even analyzed in the context of learning and teaching. Hofer et al. revealed in their first 30 abdominal free tissue breast reconstruction complications in 40% of cases. In their next 145 cases, complications decreased to 13.8%. Total revision rate was 4% and total flap failure rate was 0.6% [19]. Busic et al. reported outcomes before and after implementation of modifications gathered from an external institution, which was well experienced in microsurgical breast reconstruction [21]. Thereby, flap loss rate decreased from 9.5% to 0%, partial flap loss from 31% to 0%, revision rate from 16.6% to 9%, incidence of fat necrosis from 16.6% to 4.3%, and donor site complications from 24% to 0%. Of note, data were based on 23 free flaps only. With respect to secondary refinement procedures after DIEP flap, Enajat et al. reported outcomes of 326 patients and revealed necessity for refinements in 73% of cases [25]. Importantly, number of refinement procedures is influenced by many factors and do not necessarily mirror the quality of the initial procedure. For instance, in some cases, the patient does not wish for any intervention at the contralateral breast in the first place but asks for adjustment years later. Another important fact that needs to be pointed out in this context is reimbursement for refinement procedures. It might be possible that in health care systems in which refinement procedures are not covered, incidence and indication for such are significantly lower.

In our study, breast reconstruction with the DIEP flap led to overall complications in 12% of cases including partial flap loss in two (2%), total flap loss in one (1%), hematoma in two (2%), infections in one (1%), and fat necrosis in four cases (4%). Refinements were undertaken in 56% of patients, ranging from 44 to 73% but without statistically significant differences among study groups. In this context, refinement procedures such as reconstruction of the nipple areola complex and adjusting mastopexy of the contralateral site are always planned as a second procedure in our clinic and thus were included in the number of refinement procedures. Of note, all numbers were still well in the range of the aforementioned studies.

The same equated for complications after TUG flap breast reconstruction. Bodin et al. showed flap complications in 23% of cases, including one case of total flap loss and five cases of fat necrosis, as well as donor site-related complications in 20% of cases [26]. Craggs et al. reported flap loss in 4% of cases and donor site complications in 59% of cases [26]. The latter mostly involved wound dehiscence and infections, and were not further analyzed regarding necessity for surgical revision. Furthermore, refinements in terms of one or more sessions of fat grafting was necessary in 61% of cases [27].

In our study, one TUG flap failed, and the overall complication rate was 15%. Wound healing at the donor site was impaired in 9% of cases. Overall, our data are well in line with reports in current literature and substantiate the safe and secure approach of teaching microsurgical breast reconstruction with DIEP and TUG flaps.

This study had several limitations. Each trainee already had a vast experience in microsurgery before starting the presented training program. To find such experienced trainees might be difficult in some facilities, and this vast experience is probably not necessary for starting such a training program in most cases. However, all trainees should be confident with their microsurgical skills, irrespective of the number of cases operated on. In this context, the number of free flap procedures of each trainee prior to starting the training program varied between 52 and 198 cases, thus clearly demonstrating that some trainees required more and some less exposure to reach the highest quality of microsurgery. In addition, progression from passive to active training was based solely on subjective criteria and decided by both senior surgeons as well as the trainee themselves. However, to implement such a training program, at least one senior surgeon who is well experienced in microsurgical breast reconstruction is necessary. Ideally, this senior surgeon is familiar with teaching surgical skills in general. Thereby, the senior surgeon should have a feeling for the capacities of each individual trainee and when to approach the next step of training. Another important limitation of this study is that patient reported outcomes, such as the Breast Q [28,29,30], were not utilized to assess safety and efficacy of the presented training program. However, outcomes focused on surgical complications in the early phase after surgery and long-term follow-up provided information on the number of refinement surgeries that were all driven by the patient. Therefore, the utilized outcome measures should suffice to rate the quality of the procedures and thus safety and effectiveness of the program.

## 5. Conclusions

The introduced stepwise training approach is a safe and effective method for teaching microsurgical breast reconstruction with means of DIEP and TUG flaps. Importantly, prior to starting microsurgical breast reconstruction training, trainees must be confident with general breast surgery and microsurgery. Of note, number of training procedures and outcomes did not differ between perforator DIEP and non-perforator TUG flaps, highlighting the importance of teaching flap inset at the recipient site for an appropriate outcome.

## Figures and Tables

**Table 1:** Epidemiologic data and previous oncologic therapy of patients divided into each group and in total.

Group	Passive Training	Active Training	After Training	No-Training
Total (*n*)	34	51	59	45
DIEP (*n*)	17	24	37	18
Per trainee	2.8	4		
Range	2–4	2–7		
Age (years)	48.3	51.8	49.9	48.9
*p*-value	0.84	0.33	0.74	
DIEP (%) (vs. MS-TRAM)	82	58	89	83
*p*-value	0.94	0.09	0.55	
BMI	29.8	30.9	30.8	31.6
*p*-value	0.90	0.95	0.89	
Smoking (*n*)	0	3	0	1
*p*-value	0.92	0.49	0.81	
Hypertension (*n*)	2	2	1	3
*p*-value	0.91	0.82	0.70	
Diabetes (*n*)	0	1	0	3
*p*-value	0.66	0.79	0.40	
Radiotherapy (%)	41	92	65	72
*p*-value	0.07	0.10	0.59	
Chemotherapy (%)	41	75	51	56
*p*-value	0.41	0.19	0.77	
Immediate (%)	12	12	11	6
*p*-value	0.2	0.2	0.15	
NSM (%)	0.72	0.65	0.7	
*p*-value	0.2	0.2	0.15	
TUG (*n*)	17	27	22	27
per trainee	2.8	4.5		
Range	2–7	2–9		
Age (years)	48.2	45.8	40.5	41.7
*p*-value	0.06	0.15	0.68	
BMI	24.9	24.0	23.0	24.3
*p*-value	0.90	0.99	0.81	
Smoking (*n*)	2	2	2	0
*p*-value	0.70	0.78	0.55	
Hypertension (*n*)	0	1	3	3
*p*-value	0.45	0.56	0.89	
Diabetes (*n*)	0	0	0	1
*p*-value	0.89	0.89	0.70	
Radiotherapy (%)	53	33	45	52
*p*-value	0.95	0.18	0.66	
Chemotherapy (%)	59	43	50	56
*p*-value	0.84	0.06	0.71	
Immediate (%)	47	32	45	50
*p*-value	0.72	0.65	0.7	
NSM (%)	0.72	0.65	0.7	
*p*-value	0.72	0.65	0.7	

DIEP: deep inferior epigastric artery perforator flap; MS-TRAM: muscle sparing-transverse rectus abdominis muscle flap; TUG: transverse upper gracilis flap.

**Table 2:** Complication rates and number of refinement procedures after DIEP flap given in total and per group.

Group	Passive Training	Active Training	After Training	No-Training
DIEP (*n*)	17	24	37	18
OR time (min)	331	351	338	304
*p*-value	0.29	0.07	0.15	
Complications total (%)	6	13	16	11
*p*-value	0.59	0.89	0.26	
Revisions (%)	6	8	5	0
*p*-value	0.31	0.22	0.22	
Partial flap loss (%)	6	0	3	0
*p*-value	0.31	-	0.49	
Complete flap loss (%)	0	0	3	0
*p*-value	-	-	0.49	
Fat necrosis (%)	6	4	5	0
*p*-value	0.31	0.39	0.15	
Wound dehiscence recipient site (%)	6	0	0	0
*p*-value	0.49	-	-	
Wound dehiscence donor site (%)	0	0	0	0
*p*-value	-	-	-	
Infection recipient site (%)	0	0	5	0
*p*-value	-	-	0.6	
Infection donor site (%)	0	0	0	6
*p*-value	0.4	0.4	0.4	
Hematoma recipient site (%)	0	4	3	0
*p*-value	-	0.11	0.26	
Hematoma donor site (%)	0	4	3	5
*p*-value	0.1	0.34	0.6	
Hernia donor site (%)	0	0	3	0
*p*-value	-	-	0.36	
Refinement procedures (%)	71	63	49	44
*p*-value	0.13	0.26	0.77	

DIEP: deep inferior epigastric artery perforator flap.

**Table 3:** Complication rates and number of refinement procedures after TUG flap given in total and per group.

Group	Passive Training	Active Training	After Training	No-Training
TUG (*n*)	17	27	22	27
OR time (min)	229	214	239	237
*p*-value	0.61	0.17	0.90	
Complications total (%)	6	19	23	11
*p*-value	0.57	0.45	0.08	
Revisions (%)	0	4	9	0
*p*-value	-	0.32	0.11	
Partial flap loss (%)	6	0	0	0
*p*-value	0.21	-	-	-
Complete flap loss (%)	0	4	0	0
*p*-value	-	0.32	-	
Fat necrosis (%)	6	0	0	4
*p*-value	0.74	-	-	
Wound dehiscence recipient site (%)	0	0	0	0
*p*-value	-	-	-	
Wound dehiscence donor site (%)	6	11	9	7
*p*-value	0.85	0.40	0.26	
Infection recipient site (%)	0	0	0	4
*p*-value	0.12	-	-	
Infection donor site (%)	6	4	0	0
*p*-value	0.09	0.1	-	
Hematoma recipient site (%)	6	4	9	0
*p*-value	0.09	0.1	0.07	
Hematoma donor site (%)	0	7	9	4
*p*-value				
Refinement procedures (%)	65	41	36	56
*p*-value	0.56	0.28	0.19	

TUG: transverse upper gracilis flap.

## Data Availability

Not applicable.

## References

[1] DeSantis C.E., Ma J., Gaudet M.M., Newman L.A., Miller K.D. (2019). Breast Cancer Statistics 2019. CA Cancer J. Clin..

[2] American Society of Plastic Surgeons (2018). 2018 Plastic Surgery Statistics Report.

[3] Erdmann-Sager J., Wilkins E.G., Pusic A.L., Qi J., Hamill J.B., Kim H.M., Guldbrandsen G.E., Chun Y.S. (2018). Complications and Patient-Reported Outcomes after Abdominally Based Breast Reconstruction: Results of the Mastectomy Reconstruction Outcomes Consortium Study. Plast. Reconstr. Surg..

[4] Pien I., Caccavale S., Cheung M.C., Butala P., Hughes D.B., Ligh C., Zenn M.R., Hollenbeck S.T. (2016). Evolving Trends in Autologous Breast Reconstruction: Is the Deep Inferior Epigastric Artery Perforator Flap Taking Over?. Ann. Plast. Surg..

[5] Allen R.J., Treece P. (1994). Deep inferior epigastric perforator flap for breast reconstruction. Ann. Plast. Surg..

[6] Blondeel P.N. (1999). One hundred free DIEP flap breast reconstructions: A personal experience. Br. J. Plast. Surg..

[7] Macadam S.A., Bovill E.S., Buchel E.W., Lennox P.A. (2017). Evidence-Based Medicine: Autologous Breast Reconstruction. Plast. Reconstr. Surg..

[8] Nelson J.A., Guo Y., Sonnad S.S., Low D.W., Kovach S.J., Wu L.C., Serletti J.M. (2010). A Comparison between DIEP and muscle-sparing free TRAM flaps in breast reconstruction: A single surgeon’s recent experience. Plast. Reconstr. Surg..

[9] Selber J.C., Nelson J., Fosnot J., Goldstein J., Bergey M., Sonnad S.S., Serletti J.M. (2010). A prospective study comparing the functional impact of SIEA, DIEP, and muscle-sparing free TRAM flaps on the abdominal wall: Part I. unilateral reconstruction. Plast. Reconstr. Surg..

[10] Atisha D., Alderman A.K. (2009). A systematic review of abdominal wall function following abdominal flaps for postmastectomy breast reconstruction. Ann. Plast. Surg..

[11] Hangge P.T., Jogerst K., Mohsen A., Kosiorek H., Cronin P.A., Stucky C.H., Wasif N., Gray R.J., Rebecca A.M., Casey W.J. (2019). Making an informed choice: Which breast reconstruction type has the lowest complication rate?. Am. J. Surg..

[12] Kronowitz S.J., Robb G.L. (2009). Radiation therapy and breast reconstruction: A critical review of the literature. Plast. Reconstr. Surg..

[13] Aliu O., Zhong L., Chetta M.D., Sears E.D., Ballard T., Waljee J.F., Chung K.C., Momoh A.O. (2017). Comparing Health Care Resource Use between Implant and Autologous Reconstruction of the Irradiated Breast: A National Claims-Based Assessment. Plast. Reconstr. Surg..

[14] Nelson J.A., Voineskos S.H., Qi J., Kim H.M., Hamill J.B., Wilkins E.G., Pusic A.L. (2019). Elective Revisions after Breast Reconstruction: Results from the Mastectomy Reconstruction Outcomes Consortium. Plast. Reconstr. Surg..

[15] Razdan S.N., Cordeiro P.G., Albornoz C.R., Ro T., Cohen W.A., Mehrara B.J., McCarthy C.M., Disa J.J., Pusic A.L., Matros E. (2016). Cost-Effectiveness Analysis of Breast Reconstruction Options in the Setting of Postmastectomy Radiotherapy Using the BREAST-Q. Plast. Reconstr. Surg..

[16] Disa J.J., Cordeiro P.G., Hidalgo D.A. (1999). Efficacy of conventional monitoring techniques in free tissue transfer: An 11-year experience in 750 consecutive cases. Plast. Reconstr. Surg..

[17] Salgarello M., Pagliara D., Rossi M., Visconti G., Barone-Adesi L. (2018). Postoperative Monitoring of Free DIEP Flap in Breast Reconstruction with Near-Infrared Spectroscopy: Variables Affecting the Regional Oxygen Saturation. J. Reconstr. Microsurg..

[18] Salgarello M., Pagliara D., Rossi M., Visconti G., Barone-Adesi L. (2018). Tissue Oximetry Monitoring for Free Deep Inferior Epigastric Perforator Flap Viability: Factors to be Considered toward Optimizing Postoperative Outcome. J. Reconstr. Microsurg..

[19] Hofer S.O., Damen T.H., Mureau M.A., Rakhorst H.A., Roche N.A. (2007). A Critical Review of Perioperative Complications in 175 Free Deep Inferior Epigastric Perforator Flap Breast Reconstructions. Ann. Plast. Surg..

[20] Hallock G.G. (2008). Is There a “Learning Curve” for Muscle Perforator Flaps?. Ann. Plast. Surg..

[21] Busic V., Das-Gupta R., Mesic H., Begic A. (2006). The deep inferior epigastric perforator flap for breast reconstruction, the learning curve explored. J. Plast. Reconstr. Aesthet. Surg..

[22] Grinsell D.G., McCoubrey G.W., Finkemeyer J.P. (2016). The Deep Inferior Epigastric Perforator Learning Curve in the Current Era. Ann. Plast. Surg..

[23] Hirche C., Kneser U., Xiong L., Wurzer P., Ringwald F., Obitz F., Fischer S., Harhaus L., Gazyakan E., Kremer T. (2016). Microvascular free flaps are a safe and suitable training procedure during structured plastic surgery residency: A comparative cohort study with 391 patients. J. Plast. Reconstr. Aesthet. Surg..

[24] Kotsougiani D., Ringwald F., Hundepool C.A., Neubrech F., Kremer T., Bickert B., Kneser U., Hirche C. (2017). Safety and Suitability of Finger Replantations as a Residency Training Procedure: A Retrospective Cohort Study with Analysis of the Initial Postoperative Outcomes. Ann. Plast. Surg..

[25] Craggs B., Vanmierlo B., Zeltzer A., Buyl R., Haentjens P., Hamdi M. (2014). Donor-Site Morbidity following Harvest of the Transverse Myocutaneous Gracilis Flap for Breast Reconstruction. Plast. Reconstr. Surg..

[26] Bodin F., Dissaux C., Dupret-Bories A., Schohn T., Fiquet C., Bruant-Rodier C. (2016). The transverse musculo-cutaneous gracilis flap for breast reconstruction: How to avoid complications. Microsurgery.

[27] Enajat M., Smit J.M., Rozen W.M., Hartman E.H., Liss A., Kildal M., Audolfsson T., Acosta R. (2010). Aesthetic refinements and reoperative procedures following 370 consecutive DIEP and SIEA flap breast reconstructions: Important considerations for patient consent. Aesthetic Plast. Surg..

[28] Pusic A.L., Klassen A.F., Scott A.M., Klok J.A., Cordeiro P.G., Cano S.J. (2009). Development of a new patient-reported outcome measure for breast surgery: The BREAST-Q. Plast. Reconstr. Surg..

[29] Cohen W.A., Mundy L.R., Ballard T.N., Klassen A., Cano S.J., Browne J., Pusic A.L. (2016). The BREAST-Q in surgical research: A review of the literature 2009–2015. J. Plast. Reconstr. Aesthet. Surg..

[30] Pagliara D., Albanese R., Storti G., Barone-Adesi L., Salgarello M. (2018). Patient-reported Outcomes in Immediate and Delayed Breast Reconstruction with Deep Inferior Epigastric Perforator Flap. Plast. Reconstr. Surg. Glob. Open.

